# Persistence of Hepatitis C RNA in Liver Allografts Is Associated with Histologic Progression Independent of Serologic Viral Clearance

**DOI:** 10.1155/2009/297528

**Published:** 2009-05-05

**Authors:** M. Ghabril, R. C. Dickson, M. Krishna, R. Lloyd, J. Aranda-Michel, A. Keaveny, R. Satyanarayana, H. Bonatti

**Affiliations:** ^1^Department of Gastroenterology and Hepatology, Clarian/Indiana University School of Medicine, 975 W Walnut Street 1B327, Indianapolis, IN 46202-5181, USA; ^2^Department of Gastroenterology and Hepatology, Mayo Clinic, 4500 San Pablo Road, Jacksonville, FL 32224, USA; ^3^Department of Pathology, Mayo Clinic, 4500 San Pablo Road, Jacksonville, FL 32224, USA; ^4^Department of Pathology, Mayo Clinic, 200 First Street SW, Rochester, MN 55902-3219, USA; ^5^Department of Surgery, University of Virginia, P.O. Box 800709 Charlottesville, VA 22906, USA

## Abstract

*Background*. Hepatitis C virus (HCV) nondetectability in the liver may predict a sustained viral response (SVR) in patients with an end of treatment response. HCV RNA can be detected in liver tissue by in situ hybridization (ISH). *Aim*. To determine if HCV nondetectability in liver allografts by ISH can predict SVR in patients who cleared virus serologically on treatment. *Methods*. Twenty five patients with undetectable serum HCV on Interferon/Ribavirin therapy for HCV recurrence post liver transplant (LT) were studied. All had biopsies at 4 months post LT (baseline) and follow up with HCV ISH analysis performed. *Results*. 10 were ISH positive (group 1); 15 were ISH negative (group 2). Groups 1 and 2 had similar patient, donor, and viral characteristics at LT, as well as treatment duration at the time of the ISH assayed liver biopsy (13 ± 16 versus 10 ± 4 months *P* = .24). However, group 1 had longer total treatment duration (24 ± 10 versus 14 ± 5 months, *P* = .001). Eight (80%) group 1 and 9 (60%) group 2 patients achieved SVR. Mean grade and stage (modified Ishak score) were similar at 4 months, however, group 1 had higher grade (3 ± 1.7 versus 1.6 ± 1.3, *P* = .039) and stage (1.4 ± 1.4 versus 0.5 ± 0.6, *P* = .084) on the ISH assayed biopsy, after similar post LT intervals (23 ± 10 versus 24 ± 12 months, *P* = .91). *Conclusion*. Allograft HCV ISH nondetectability does not predict SVR in treatment responsive HCV recurrence, but is associated with less severe histologic disease.

## 1. Introduction

Hepatitis C virus (HCV) is the most common indication for liver transplantation (LT) in the US with almost universal recurrence following LT. The need for antiviral therapy is common with up to 30% of patients progressing to cirrhosis by 5 years [1]. Though the indication for antiviral therapy is based on histologic findings, the primary goal of treatment goal is serologically defined by a sustained viral response (SVR).

The absence of HCV RNA in liver tissue at the end of treatment has been associated with SVR in HCV patients with chronic liver disease treated with interferon-based antiviral therapy [2]. A recently reported series in HCV LT patients suggested that the presence or absence of allograft HCV RNA following treatment predicted a relapse or SVR in patients with a loss of viremia at the end of treatment. All 7 patients with a negative hepatic HCV RT-PCR at the end of treatment had an SVR while 3 patients with HCV RT-PCR present had relapse [3]. 

However, SVR status has not uniformly resulted in histologic stabilization or fibrosis regression. It has been observed in a previous report that twenty percent of post LT HCV patients experienced fibrosis progression 3–5 years following SVR [4, 5], while fibrosis regression has been described in treated patients without SVR [6]. Additionally, hepatic HCV RNA persistence has been described in patients with SVR [7]. The correlation of hepatic allograft HCV RNA detectability post LT with serum virologic endpoints and histologic outcomes remains uncertain.

HCV RNA can be detected in liver tissue by polymerase chain reaction (PCR), immunohistochemical, and in situ hybridization (ISH) assays. HCV ISH utilizes digoxigenin labeled riboprobes with sense (genomic) and antisense (replicative intermediate) transcripts corresponding to the 5′ noncoding region of the virus. It can be performed on paraffin-embedded sections, with positive and negative controls, and yields a qualitative result. It has been shown to correlate well with tissue HCV RNA detection by PCR [8].

The primary aim of this study was to determine if HCV nondetectability in liver allografts by ISH can predict SVR in patients who cleared virus serologically on antiviral treatment. A secondary aim was to determine the correlation of allograft HCV-ISH status with histologic disease in this patient subset.

## 2. Patients and Methods

We retrospectively reviewed the records of HCV patients undergoing LT at our center between February 1998 and December 2005. The HCV ISH assay became available at our institution in January 2003. It was performed in selected patients with undetectable serum HCV by PCR while receiving antiviral therapy. These patients were further analyzed. Data was retrospectively collected through April 2007. The study was approved by the study centers Institutional Review Board.

The ISH assay was performed as previously described [8]. Patients were grouped by HCV ISH status (positive or negative), and compared for (1) patient and donor characteristics. (2) Antiviral therapy and virologic outcomes of early viral response (EVR), end of treatment response (ETR) and SVR. SVR was defined as undetectable HCV RNA in serum by qualitative assay ≥6 months after completion of antiviral therapy. (3) Histologic findings of grade and stage at baseline 4 months post LT (protocol biopsy) and at the ISH assayed biopsy. Grading and staging of all biopsies were performed using the modified Ishak score by a single pathologist (MK) who was blinded to the ISH result, serum viral status, and clinical findings [9].

All LT procedures were performed using piggy back technique. Initial immunosuppression included a 3 drug regimen of tacrolimus, mycophenolate mofetil and prednisone, and tacrolimus monotherapy after 4 months post LT. Protocol liver biopsies were performed at 7 days, 4 months, and annually post LT, and as clinically indicated. Antiviral therapy using Interferon (interferon alfa 2b prior to 2002 and peginterferon alfa 2a since 2002) and ribavirin was initiated for significant HCV recurrence. HCV recurrence was defined as liver enzyme elevation ≥2× the upper limits of normal, detectable serum HCV RNA, and histologic features consistent with hepatitis C with Batts Ludwig activity score of ≥2 and/or progressive fibrosis. Minimum planned duration of therapy was 48 weeks for genotypes 1 and 4, and 24 weeks for genotypes 2 and 3. Growth factors were used as clinically indicated. Prior to 2005 serum HCV detectability was determined by the Roche COBAS Amplicor HCV Monitor 2.0 assay (Roche Diagnostics, Indianapolis, IN) with a lower limit of detection of 50 IU/mL. Since 2005 serum HCV was assayed with the COBAS TaqMan HCV Test (TaqMan HCV; Roche Molecular SystemsInc., Branchburg, N.J.) with a dynamic range of 10 IU/mL to 50,000,000 IU/mL.

## 3. Statistical Analysis

Patient groups were compared using the Mann-Whitney and chi-square tests. SPSS 14.0 (SPSS Inc., Chicago, IL) was used for the analysis. All *P* values were 2 tailed, and *P* < .05 was considered statistically significant. All values are shown as mean ± 1 SD, or percentage unless otherwise specified.

## 4. Results

LT for HCV were performed in 460 patients between 1998 and 2005 at our center and 231 (50%) underwent antiviral treatment. Of the treated patients 73 (31.6%) had an on treatment response, 44 had an SVR, 16 relapsed and 13 remained on treatment. HCV ISH assays of liver biopsies were performed prospectively in 26/73 (36%) of the patients with undetectable serum HCV by PCR while on treatment between July 2004 and June 2006. Ten patients were ISH positive (group 1), 15 were ISH negative (group 2), and 1 was excluded due to indeterminate ISH results. Serum HCV was not detectable at the time of ISH assayed liver biopsies based on the highly sensitive COBAS Taqman serum HCV RNA assay in 22 (88%) of the 25 patients since 2005, and based on the COBAS Amplicor HCV Monitor assay in 3 (12%) patients before 2005.

Groups 1 and 2 were similar for patient, donor, and viral characteristics, with the exception of a trend toward more female patients in group 1 (Table 1). Antiviral therapy timing, duration at the time of the ISH assayed biopsy, treatment tolerance, and virologic outcomes were similar for groups 1 and 2, with the exception of longer total treatment duration in group 1 (Table 2). All patients received at least the minimum planned duration of treatment per genotype. Eight (80%) group 1 patients achieved SVR, 1 (10%) relapsed and 1 (10%) remained on therapy with undetectable serum HCV at last follow up. Nine (60%) group 2 patients achieved SVR, and 6 (40%) relapsed.

Antiviral treatment outcomes collated by patient group, HCV genotype, duration of treatment at the time of ISH assayed biopsy, total treatment duration, and timing of virologic response are described for each case in Table 3. After 12 weeks of therapy, HCV RNA was undetectable by qualitative assay in 11 patients, and detectable in 8 (5 of whom had EVR). A qualitative assay at 12 weeks was not performed in 6 patients (5 of whom had EVR). Eight of the 11 patients with undetectable serum HCV by 12 weeks of therapy achieved SVR with treatment of at least the minimum planned duration per genotype, while 2 relapsed, and 1 remained on therapy for low levels of detectable HCV (<10 IU/mL) after >6 months of therapy. Seven of the 8 patients with detectable HCV at 12 weeks had undetectable HCV by week 24. Four of these 7 patients received ≥24 weeks of additional therapy beyond their minimum planned treatment duration per genotype, 3 achieved SVR, and 1 relapsed. The other 3 patients received <24 weeks of additional therapy, and all 3 relapsed. One patient with detectable serum HCV at week 24 became undetectable at 19 months, and achieved SVR after a total of 33 months of therapy.

ALT, histologic grade, and stage were similar for the 2 groups at 4 months post LT; however, at the time of the follow up ISH biopsy they were higher in group 1 patients (Table 4). There were no deaths during the study period.

## 5. Discussion

The data from this study supports two primary findings. First the absence of allograft HCV RNA by ISH did not predict SVR in patients with an ETR. Second, there was a correlation of liver allograft HCV RNA detectability by ISH with increased disease activity and fibrosis in the liver allograft. This finding was independent of serologic viral clearance.

Viral relapse in 7 of 14 patients with undetectable hepatic HCV RNA by ISH on or at the end of completed treatment was unexpected. A lack of sensitivity of the ISH assay is one possibility. However, while there is no gold standard to define HCV detectability in liver tissue, the ISH assay has been shown to correlate well with tissue PCR techniques and immunohistochemical assays [8]. Sampling variation has been described in the grading and staging of liver biopsies specimens in patients with chronic HCV [10]. Thus, this could also account for these findings as the ISH assay was taken from only a single liver biopsy specimen. Extrahepatic compartmentalization of HCV has been well described and may theoretically explain these contradictory findings [7, 11]. Extrahepatic sources may provide alternative viral reservoirs and account for reinfection of the liver following HCV RNA clearance from the liver and serum while on treatment. The concept of extrahepatic reservoirs of hepatotropic viruses has been well described with Hepatitis B virus (HBV). HBV DNA has been found in serum and lymphocytes many years following successful LT despite no clinical evidence of HBV recurrence [12, 13].

Virologic relapse in those with an ETR and undetectable hepatic HCV by ISH post LT in our series is at odds with the finding of SVR in all 7 patients with an ETR and undetectable hepatic HCV by PCR post LT in a prior study [3]. Both series suffer from small sample size, but a difference in the assay of hepatic HCV may account for the difference. ISH involves in situ detection of viral RNA in hepatocytes, whereas PCR analysis of homogenized liver tissue would include some circulating lymphocytes harboring virus.

Our series comprised a select group including only patients with loss of HCV serum RNA on treatment, thus the higher than expected SVR rates were anticipated. There was a statistically insignificant but numerically higher rate of SVR in the ISH positive versus negative group, 80% versus 60%, respectively. However, the differences were most likely due to the longer duration of antiviral treatment given and a higher percentage of genotypes 2 or 3 in patients with positive ISH (44% versus 14%). Longer duration of therapy in group 1 patients was attributed to a treatment bias based on the finding of positive HCV ISH, and likely reduced relapse rates in the subset of patients with EVR, detectable HCV at 12 weeks, and undetectable HCV at 24 weeks [14, 15]. Interestingly, the patient with detectable serum HCV at 24 weeks cleared virus late into treatment and achieved SVR.

The second major finding of the study was the correlation of hepatic HCV RNA detectability by ISH with increased histologic activity despite similar demographic factors in the ISH positive and negative groups. The presence of HCV RNA in native liver tissue has been associated with increased histologic necroinflammatory activity and fibrosis in patients with chronic liver disease secondary to HCV [16–19]. Post LT, Neff et al. noted that 6 of 7 patients with detectable hepatic HCV by RT-PCR had grade 1-2 inflammation at the end of treatment while in 3 of 4 patients with no inflammation the HCV RT-PCR in the liver was undetectable [3]. Our data may suggest an important role of hepatic HCV RNA in eliciting or maintaining an immune response regardless of a loss of HCV RNA in the serum. Allograft histologic progression has been described in 20% of patients three to five years following serologic SVR [4, 5], perhaps this could be accounted for by remnant hepatic HCV RNA.

In summary, this is a retrospective study and is limited by sample size, but it suggests that hepatic HCV detectability has limited value for predicting sustained virologic response in post LT HCV patients achieving loss of HCV RNA on current antiviral therapy. The correlation of hepatic HCV RNA with histologic activity represents a preliminary finding which merits further investigation.

## Figures and Tables

**Table 1:** Patient and donor characteristics, and antiviral therapy at the time the ISH biopsy in HCV ISH positive (group 1) and negative (group 2) patients.

Variables	Group 1 ISH positive *N* = 10	Group 2 ISH negative *N* = 15	*P*
Patient age at LT	49 ± 11	52 ± 11	.66
Female gender (patient)	40%	13%	.13
Caucasian (patient)	90%	87%	.69
Recipient body mass index	31 ± 7	29 ± 6	.51
MELD at LT	14 ± 5	18 ± 9	.42
Genotype 1 (unknown in 1 patient per group)	56%	86%	.11
Donor age	47 ± 13	43 ± 14	.82
Female gender (donor)	40%	53%	.51
Caucasian (Donor)	90%	67%	.34
Cold ischemia time, hours	7 ± 2	7 ± 2	.74
Warm ischemia time, minutes	39 ± 9	34 ± 10	.28
Tacrolimus as primary immune suppressant	100%	83%	.23
Steroid bolus treated ACR	30%	40%	.61
LT to ISH biopsy interval, months	23 ± 10	24 ± 12	.91

**Table 2:** Antiviral therapy timing, duration, dose reductions, growth factor use, and virologic outcomes in treated group 1 and 2 patients.

Variable	Group 1 ISH positive *N* = 10	Group 2 ISH negative *N* = 15	*P*
Pegylated IFN	100%	86%	.23
Dose reduction	40%	33%	.73
Growth factors used	80%	73%	.70
LT-Treatment interval, months	6 ± 3	9 ± 7	.35
Treatment duration at ISH biopsy months	13 ± 6	10 ± 4	.24
Total treatment duration	24 ± 10	14 ± 5	.001
EVR	90%	86%	.75
ETR	90%*	100%	NA
SVR	80%*	60%	.29

*1 patient was still on treatment at last follow up.

**Table 3:** Antiviral treatment duration and outcomes collated by patient group, HCV genotype, timing of ISH assayed biopsy and virologic response.

Group, genotype and case number	ISH Status	Treatment duration at ISH assay (months)	Total treatment duration (months)	Viral load response	HCV undetectable by 12 weeks	HCV undetectable by 24 weeks	Outcome
Group 1, *genotype 2 or 3*							

1	+	12	12	EVR	No	Yes	SVR
2	+	12	18	EVR	NA	Yes	SVR
3	+	21	26	RVR	Yes		SVR
4	+	14	37	EVR	Yes		On therapy

Group 1, genotype 1 or unknown*							

5	+	4	18	EVR	No	Yes	Relapse
6	+	5	19	EVR	Yes		SVR
7*	+	9	18	EVR	Yes		SVR
8	+	14	21	No EVR	No	Yes	SVR
9	+	21	46	EVR	NA	Yes	SVR
10	+	13	22	EVR	Yes		SVR

Group 2, *genotype 2 or 3*							

11	−	7	11	RVR	Yes		SVR
12	−	9	9	No EVR	No	Yes	Relapse

Group 2, genotype 1 or unknown*							

13	−	10	11	EVR	NA	Yes	SVR
14	−	8	12	EVR	NA	Yes	Relapse
15	−	6	11	RVR	Yes		SVR
16*	−	8	12	EVR	Yes		SVR
17	−	10	13	EVR	Yes		Relapse
18	−	14	14	EVR	NA	Yes	SVR
19	−	11	12	EVR	No	Yes	Relapse
20	−	20	33	No EVR	No	No	SVR
21	−	3	12	EVR	Yes		SVR
22	−	14	14	NA	NA	Yes	SVR
23	−	7	12	EVR	No	Yes	Relapse
24	−	13	17	EVR	No	Yes	SVR
25	−	12	13	EVR	Yes		Relapse

NA: Not available; EVR: Early virologic response; RVR: Rapid virologic response; SVR: Sustained viral response.

*The patients that did not have a known genotype.

**Table 4:** HCV viral loads, ALT, and modified Ishak score grade and stage for groups 1 and 2, at 4 months post LT and at the ISH biopsy.

Variables	Group 1 ISH positive *N* = 10	Group 2 ISH negative *N* = 15	*P*
4 month HCV titer x1,000,000 IU/ml	1.2 ± 15	2.4 ± 4.4	.32
4 month ALT IU	126 ± 92	168 ± 205	.68
4 month grade	3.6 ± 2.5	3.6 ± 2.7	.9
4 month stage	1 ± 1.2	0.5 ± 0.5	.39
4 month to ISH biopsy interval, months	19 ± 10	19 ± 12	.93
ISH biopsy ALT IU	69 ± 45	32 ± 27	.016
ISH biopsy grade	3 ± 1.7	1.6 ± 1.3	.039
ISH biopsy stage	1.4 ± 1.4	0.5 ± 0.6	.084

## Abbreviations

HCV: Hepatitis C virus
LT: Liver transplantation
ISH: In situ hybridization
EVR: Early viral response
ETR: End of treatment response
SVR: Sustained viral response
PCR: Polymerase chain reaction.
